# Real-Time Quaking-Induced Conversion Detection of PrP^Sc^ in Fecal Samples From Chronic Wasting Disease Infected White-Tailed Deer Using Bank Vole Substrate

**DOI:** 10.3389/fvets.2021.643754

**Published:** 2021-03-04

**Authors:** Soyoun Hwang, Justin J. Greenlee, Eric M. Nicholson

**Affiliations:** Virus and Prion Research Unit, National Animal Disease Center, United States Department of Agriculture, Agricultural Research Service, Ames, IA, United States

**Keywords:** CWD, prion disease, RT-QuIC, transmissible spongiform encephalopathy, TSE, feces, white-tailed deer

## Abstract

Chronic wasting disease (CWD) is a transmissible spongiform encephalopathy (TSE) that is fatal to free-range and captive cervids. CWD has been reported in the United States, Canada, South Korea, Norway, Finland, and Sweden, and the case numbers in both wild and farmed cervids are increasing rapidly. Studies indicate that lateral transmission of cervids likely occurs through the shedding of infectious prions in saliva, feces, urine, and blood into the environment. Therefore, the detection of CWD early in the incubation time is advantageous for disease management. In this study, we adapt real-time quacking-induced conversion (RT-QuIC) assays to detect the seeding activity of CWD prions in feces samples from clinical and preclinical white-tailed deer. By optimizing reaction conditions for temperature as well as the salt and salt concentration, prion seeding activity from both clinical and preclinical animals were detected by RT-QuIC. More specifically, all fecal samples collected from 6 to 30 months post inoculation showed seeding activity under the conditions of study. The combination of a highly sensitive detection tool paired with a sample type that may be collected non-invasively allows a useful tool to support CWD surveillance in wild and captive cervids.

## Introduction

Chronic wasting disease (CWD) is a form of transmissible spongiform encephalopathy (TSE) or prion disease affecting cervids including deer, elk, reindeer, and moose. Prion diseases result from the misfolding of the cellular prion protein (PrP^C^) into a pathogenic form (PrP^Sc^). Other prion diseases include scrapie in sheep, bovine spongiform encephalopathy (BSE) in cattle, and Creutzfeldt-Jakob disease (CJD), fatal familial insomnia (FFI), Gerstmann-Sträussler-Scheinker syndrome (GSS), and kuru in humans. CWD has been reported across North America including 26 states in the United States and three Canadian provinces. CWD-infected animals have also been reported in South Korea, Norway, Finland, and most recently, Sweden (1–4).

Chronic wasting disease infected cervids have misfolded prion proteins distributed widely, not only in the nervous system but also in lymphoid tissues, muscle, and blood (5–7). These animals are known to shed prions into the environment *via* saliva, urine, blood, and feces. This environmental contamination is often suggested to be the cause for horizontal CWD transmission among captive and free-ranging wild animals. Despite the awareness of potential sources, rising case numbers in wild cervids highlight the lack of effective CWD management strategies. Lacking treatment or vaccination, any management strategy for CWD will be dependent on sensitive and early detection of CWD prions in CWD infected animals. Early detection in a readily accessible sample that can be collected in a non-invasive procedure will afford producers and regulatory entities the opportunity to test prior to shipment as well as upon receipt to reduce the likelihood that an infected animal would be introduced into an otherwise healthy herd. Samples that are shed from animals, particularly early in the incubation period, have low concentrations of detectable prions, which necessitates highly sensitive prion detection methods. Highly sensitive prion detection tools like real-time quaking induced conversion (RT-QuIC) that amplify the prion *in vitro* enabled the detection of prions from early stage of the disease and from various sample types in addition to brain and lymphoid tissues. Several reports have indicated that fecal prions as well as those found in saliva, blood, and urine from cervids could be detected using RT-QuIC, in some cases using preclinical samples (8–14).

In the study, we tested the suitability of bank vole prion protein (BV rPrP) as a substrate for CWD detection in feces samples from clinical and preclinical white-tailed deer. To accomplish this, the reaction conditions were optimized using different enrichment methodologies, different salt concentrations, different temperatures, and different salt ions to amplify fecal prions for improved sensitivity and specificity.

## Materials and Methods

### Source of Fecal Samples

All fecal samples used in this manuscript were collected from white-tailed deer that were experimentally inoculated by the oronasal route with the CWD agent from either a white-tailed deer (deer numbers #1548 and #1553) or an experimentally inoculated raccoon (deer numbers #1542 and #1555). Feces was collected from two non-inoculated, non-CWD exposed deer for use as negative controls (deer numbers #831 and #1801). They are summarized in Table 1. All animal experiments were conducted at the National Animal Disease Center under the approval of the Institutional Animal Care and Use Committee (protocol number: ARS-2018-748, date of approval from ethical committee: August 7, 2015). The animal experiments were carried out in accordance with the Guide for the Care and Use of Laboratory Animal (Institute of Laboratory Animal Resources, National Academy of Sciences, Washington DC, USA). Each deer was inoculated oronasally similar to previously described (15) with 1 ml of 10% (wt/vol) brain homogenate from a single animal with clinical signs consistent with prion disease and confirmed positive by immunohistochemistry, enzyme-immunoassay (EIA), and Western blot. Fecal samples were collected at ~6-month intervals until the deer developed clinical signs consistent with CWD. The mean incubation period was 682 days for deer inoculated with the CWD agent from a white-tailed deer. At ~1,700 dpi, the experiment with deer inoculated with the CWD agent from raccoons is ongoing, but deer #1542 was euthanized at 1,035 dpi due to clinical signs consistent with CWD.

**Table 1:** Animal experimental summary of genotype, inoculum, survival period, and enzyme-immunoassay (EIA) in white-tailed deer inoculated oronasally with the agent of chronic wasting disease from white-tailed deer and racoon.

**Eartag**	**Genotype**	**CWD Inoculum**	**Months post- incubation (mo.)**	**^**b**^EIA O.D**.
	**96**			
1553	GG	WTD	21.4	4.00
1548	GG	WTD	24	4.00
1542	GG	Racoon CWD	34.5	4.00
1555	GG	Racoon CWD	^a^56.7	NT
831	GG	Neg. control	42	^c^0.07
1801	GG	Neg. control	34.1	^c^0.08

*NT indicates that samples were not tested*.

a *This animal has not developed clinical signs at the time of this study*.

b *IDEXX HerDCheck CWD Ag test*.

c *Values below 0.18 are negative*.

### Preparation of Cervid Feces Extracts

Cervid feces was prepared as described by Cheng et al. (9). Briefly, 1 g of previously collected fecal pellets was weighed and added into the feces extract buffer (20 mM sodium phosphate (pH 7.1), 130 mM NaCl, 0.05% Tween 20, 1 mM phenylmethylsulfonyl fluoride (PMSF), and 1X complete protease inhibitors (Roche) giving a final concentration of 10% (w/v). Then, the fecal pellets were homogenized in M tubes (GentleMACS M tubes) using a dissociator (GentleMACS, Miltenyi Biotec) for a minute with two to three repeats to ensure complete pellet disruption. The tubes were then placed onto an orbital shaker for 1 h at room temperature. After centrifugation at 18,000 × g for 5 min, supernatants were collected and stored at −80°C for further use.

### Sodium Phosphotungstic Acid Precipitation

To each 1 ml volume of 10% (w/v) fecal supernatant, 250 μl of 10% N-lauryl sarcosine was added. Samples were then incubated at 37°C for 30 min at 1,400 rpm in a thermomixer. Using a stock solution of 10% sodium phosphotungstic acid (NaPTA), 170 mM of magnesium chloride (pH 7.4) was added to each sample to give a final concentration of 0.3% NaPTA in the samples. Samples were incubated at 37°C for 2 h with shaking at 400 rpm. Supernatants were removed following centrifugation at 15,800 × g for 30 min. Pellets were washed with wash buffer [10 mM Tris-Cl, pH 7.5, 100 mM NaCl, 0.5% Triton-X 100, 10 mM ethylenediamine tetraacetic acid (EDTA), 0.5% sodium deoxycholate (w/v), and 0.1% sarkosyl (w/v)], and centrifuged again for 15 min at 15,800 g. Pellets were resuspended in 100 μl of Dulbecco's phosphate-buffered saline (DPBS) with 0.05% Sodium dodecyl sulfate (SDS).

### PAD-Beads Enrichment

PAD-Beads and all buffers were provided as a kit from (Microsens, London, UK). Standard PAD-Beads enrichment was followed as described by Hwang et al. (16). Briefly, 200 μl of 10% (w/v) fecal samples was mixed with 500 μl of distilled water by gently tapping the tubes, and then 200 μl of capture buffer and 100 μl of beads were added to the tube. The tubes were then shaken for 30 min at room temperature on a rocker. After the incubation, beads were captured on a magnet and the liquid was removed. Then, the samples were washed with wash buffer 1 and wash buffer 2. For elution, 25 μl of elution buffer (0.1 M NaOH, 0.1% Triton X-100) was added to the beads, and the tubes were shaken for 5 min. The tubes were placed on a magnet to capture the beads. While the tubes were on the magnet, the same volume, 25 μl of 0.1 M HCl was added to the tubes to neutralize the NaOH and mixed gently. Finally, the liquid was removed and analyzed by RT-QuIC.

For large scale sample preparation, 1 ml of fecal samples was used instead of 200 μl, and all other buffers were used at five times the previously indicated volume except the elution buffer and HCl neutralization which were both 50 μl.

### Recombinant Prion Protein Production and Purification

*Escherichia coli* [BL21 (λDE3)] was transformed with the pET28a vector containing the bank vole PrP gene (amino acids 23–231; GenBank accession number AF367624), and the recombinant bank vole prion proteins were expressed and purified as described by Vrentas et al. (17). The concentration of pooled protein eluent was measured by UV and calculated from the absorbance at 280 nm using an extinction coefficient of 62,005 M^−1^cm^−1^ as calculated for the bank vole prion protein (18).

### Real-Time Quaking-Induced Conversion Protocol

Real-time quaking-induced conversion reactions were performed as previously described (19–25). The reaction mix was composed of 10 mM phosphate buffer (pH 7.0), either 100, 200, 300, 400, and 500 mM NaCl or 100, 200, 300, 400, and 500 mM sodium iodide (NaI), 0.1 mg/ml recombinant bank vole prion protein, 10 μM thioflavin T (ThT), and 1 mM EDTA tetrasodium salt. Aliquots of the reaction mix (98 μL) were loaded into each well of a black 96-well plate with a clear bottom (Nunc, Thermo Fisher Scientific, USA) and seeded with 2 μL of fecal homogenate dilutions with 0.05% SDS. The plate was then sealed with plate sealer film and incubated at 37, 42, or 48°C in a BMG FLUOstar Omega plate reader with cycles of 1 min shaking (700 rpm double orbital) and 1 min rest for 100 h. ThT fluorescence measurements (excitation 460 nm, emission 480 nm, bottom read, 20 flashes per well, and manual gain 1,400) were taken every 45 min.

All RT-QuIC assays for each dilution of each sample were performed as two repeats of four replicates for a total of eight replicates. ThT fluorescence data are displayed as the average ThT fluorescence of four technical replicates for each time point and, to be considered positive, the ThT fluorescence of at least two replicates out of four reactions must be positive. Positive threshold was calculated as the mean value of non-inoculated control sheep brain homogenates plus 10 SDs, and lag time is defined as the time to reach the positive threshold (20, 26, 27).

## Results

### Real-Time Quaking-Induced Conversion Detection of PrP^Sc^ in Non-enriched Fecal Samples From White-Tailed Deer Clinically Affected With CWD

**Figure 1:**
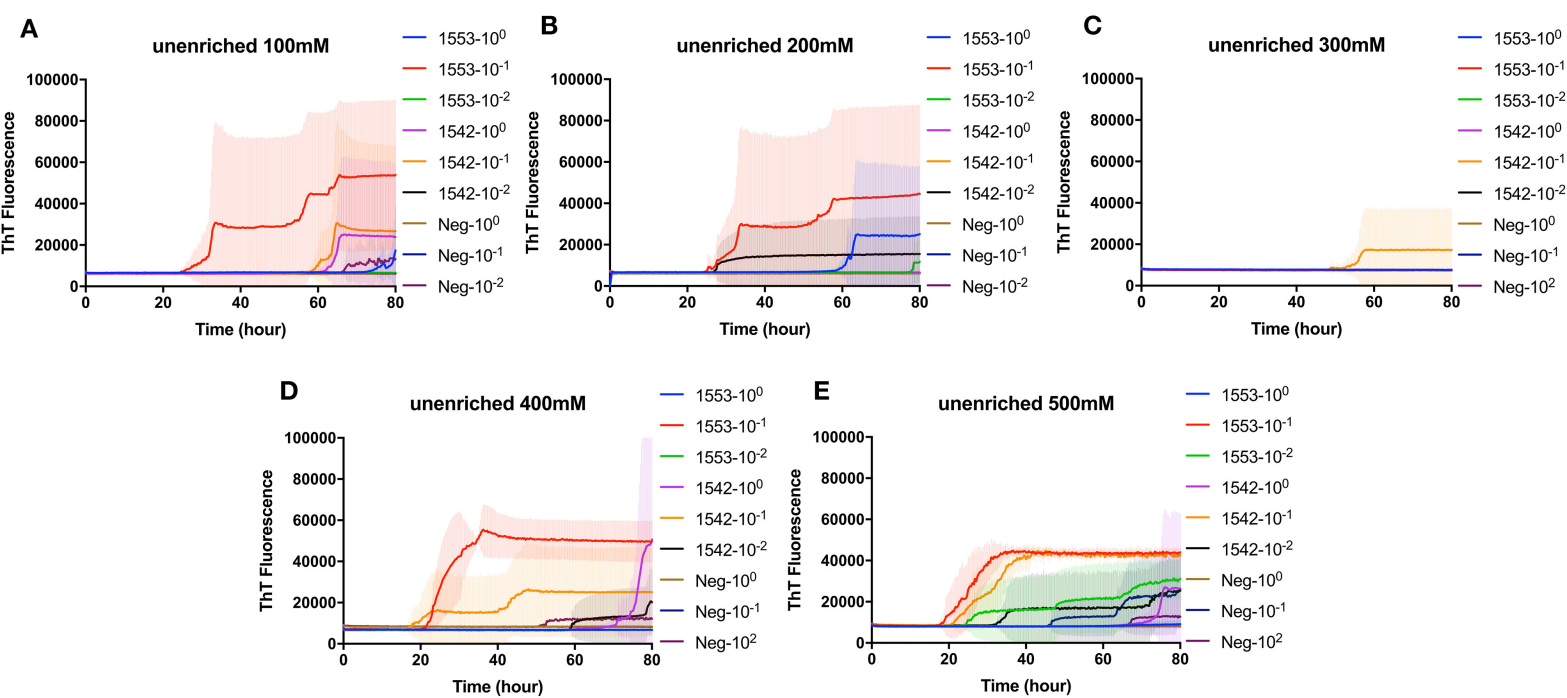
Real-time quacking-induced conversion (RT-QuIC) reactions containing different NaCl concentrations **(A)** 100 mM, **(B)** 200 mM, **(C)** 300 mM, **(D)** 400 mM, and **(E)** 500 mM seeded with fecal dilutions from chronic wasting disease (CWD) infected white-tailed deer brains using BV rPrP as a substrate. RT-QuIC reactions were seeded with 10^0^, 10^−1^, or 10^−2^ dilutions of 10% fecal homogenate. All reactions were seeded with fecal homogenate of white-tailed deer with the addition of 0.001% of SDS. Shown are the average ThT fluorescence readings determined from all replicates (four replicate reactions per each dilution).

To test whether CWD prions from fecal samples, without enrichment or substrate replacement, could be detected by RT-QuIC using BV rPrP, reactions were seeded with different dilutions of fecal samples collected from clinically affected white-tailed deer at the time of necropsy. Different concentrations of NaCl were tested to find the optimal reaction conditions with BV rPrP and CWD infected deer fecal samples (Figure 1). All tested conditions showed an increase in ThT fluorescence typical for the detection of TSE within 30 h except 300 mM NaCl. Using higher salt concentrations (400 and 500 mM NaCl) improved the seeding activity with shorter lag time for assays seeded with feces from positive animal samples while assays seeded with feces from negative control samples remain below the threshold to be considered positive (Figures 1D,E).

### Real-Time Quaking-Induced Conversion Detection of PrP^Sc^ in Enriched Fecal Samples by NaPTA Precipitation From White-Tailed Deer Clinically Affected With CWD

**Figure 2:**
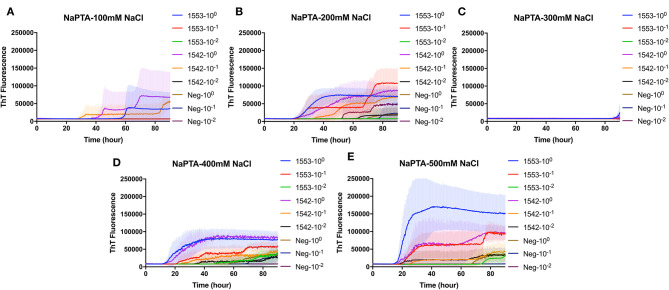
Real-time quacking-induced conversion (RT-QuIC) reactions containing different NaCl concentrations **(A)** 100 mM, **(B)** 200 mM, **(C)** 300 mM, **(D)** 400 mM, and **(E)** 500 mM seeded with sodium phosphotungstic acid (NaPTA) enriched fecal dilutions from CWD infected white-tailed deer brains using BV rPrP as a substrate. RT-QuIC reactions were seeded with 10^0^, 10^−1^, or 10^−2^ dilutions of 10% fecal homogenate. All reactions were seeded with fecal homogenate of white-tailed deer with the addition of 0.001% of SDS. Shown are the average ThT fluorescence readings determined from all replicates (four replicate reactions per each dilution).

To evaluate the efficacy of NaPTA precipitation in detection CWD prions in fecal samples from clinical CWD infected white-tailed deer, reactions containing recombinant BV rPrP were seeded with different dilutions of NaPTA enriched fecal samples. Again, different concentrations of NaCl were tested to find the optimal condition for the detection of PrP^Sc^ in the fecal samples with RT-QuIC following NaPTA precipitation. Similar to the results observed for non-enriched samples, all tested NaCl concentrations, except 300 mM NaCl, result in fibril seeding based on an observed increase in ThT fluorescence (Figure 2). Assays with higher NaCl concentrations (400 and 500 mM) showed shorter lag times with spontaneous conversion with low intensity and extended lag time at 70 h. It is clear that NaPTA precipitation improved the RT-QuIC detection in fecal samples by shortening lag time of seeding activity.

### Real-Time Quaking-Induced Conversion Detection of CWD Prions in Fecal Samples of White-Tailed Deer With Clinical Signs of CWD Following PAD-Bead Enrichment

**Figure 3:**
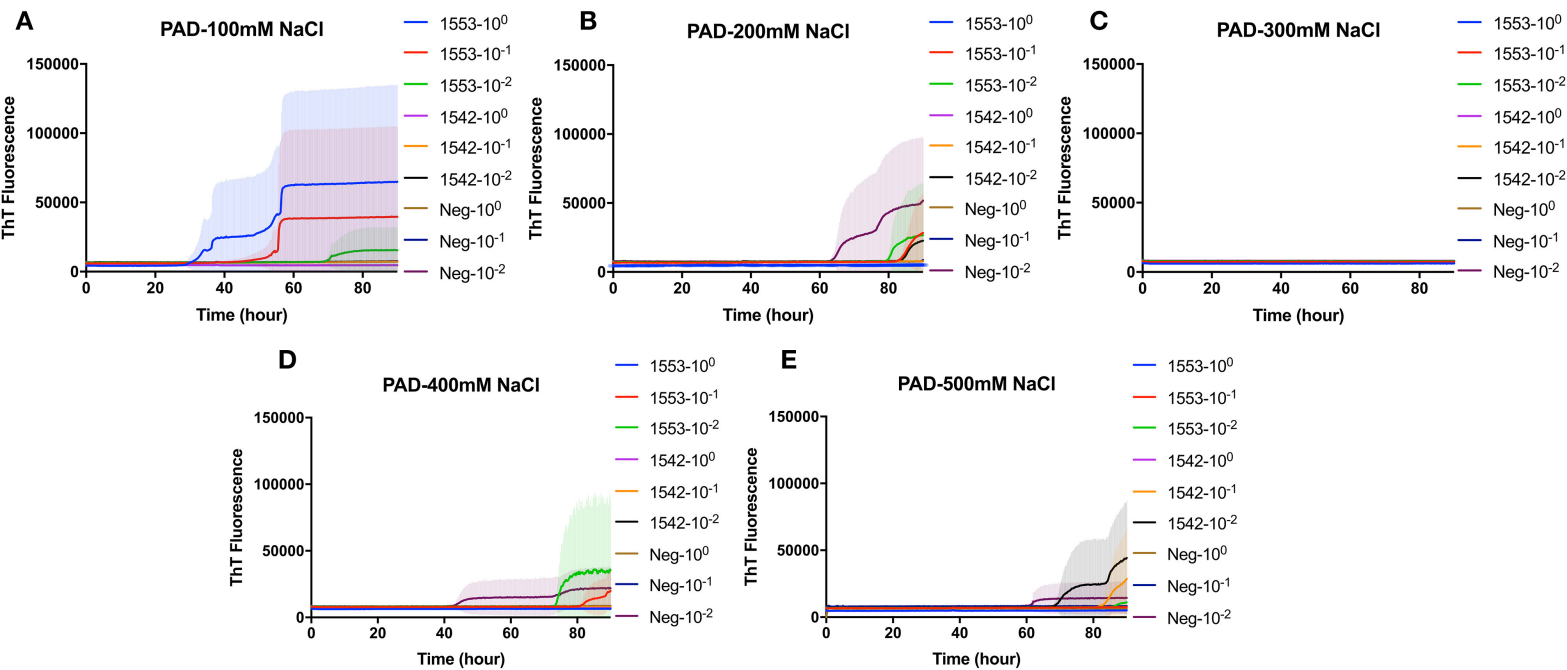
Real-time quacking-induced conversion (RT-QuIC) reactions containing different NaCl concentrations **(A)** 100 mM, **(B)** 200 mM, **(C)** 300 mM, **(D)** 400 mM, and **(E)** 500 mM seeded with PAD-Beads enriched fecal dilutions from CWD infected white-tailed deer brains using BV rPrP as a substrate. RT-QuIC reactions were seeded with 10^0^, 10^−1^, or 10^−2^ dilutions of 10% fecal homogenate. All reactions were seeded with fecal homogenate of white-tailed deer with the addition of 0.001% of SDS. Shown are the average ThT fluorescence readings determined from all replicates (four replicate reactions per each dilution).

**Figure 4:**
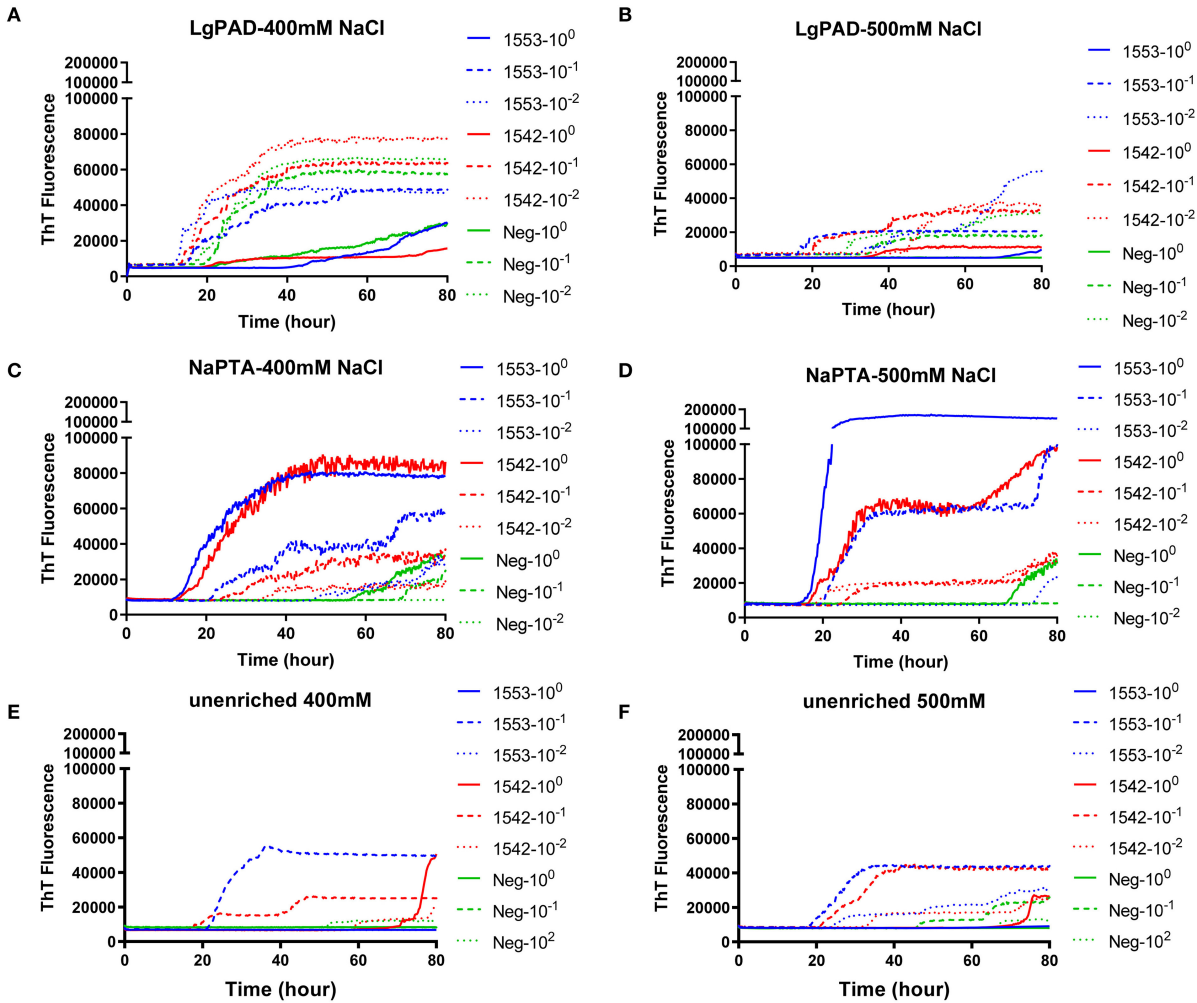
Comparison of RT-QuIC reactions seeded with fecal dilutions from different enrichment. **(A,B)** enriched with PAD-Beads large scale **(C,D)** enriched with NaPTA **(E,F)** non-enriched dilutions. All reactions were seeded with fecal homogenate of white-tailed deer with the addition of 0.001% of SDS. Shown are the average ThT fluorescence readings determined from all replicates (four replicate reactions per each dilution).

PAD-Beads, a commercially available kit, has been used to successfully enrich brain homogenate samples prior to RT-QuIC (16). To evaluate the efficacy of PAD-Beads enrichment for CWD prion detection from fecal samples, reactions containing BV rPrP were seeded with different dilutions of PAD-Beads eluate in the presence of different NaCl concentrations. Most assays did not show any increase of ThT. However, assays containing 100 mM NaCl showed the increase of ThT fluorescence for the assays seeded with fecal sample of animal #1553 (Figure 3). Given the presumably lower concentration of PrP^Sc^ in fecal samples relative to brain homogenate, we also assessed a higher starting volume of fecal sample homogenate. The standard PAD-Bead protocol uses 200 μl of 10% fecal homogenates. Therefore, instead of using 200 μl of 10% fecal homogenates (standard protocol), 1 ml of 10% fecal homogenate was used for PAD-Beads enrichment. All other reagents in the protocol were also increased by five times. At the end, 100 μl of eluate was collected to make the final 1/10 volume of original fecal sample, a volume equivalent to that used in NaPTA enrichment. When assays were seeded with fecal eluate of large scale PAD-Beads enrichment, every assay showed rapid (within 20 h) conversion. However, assays seeded the equivalently treated negative control fecal samples showed seeding activity with only a marginally longer lag time. Specifically, assays seeded with negative control in the presence of 400 mM NaCl had a lag time ~5 h longer than from positive animals, and all reactions containing 500 mM NaCl showed the increase of ThT fluorescence in a similar lag time albeit lower intensity (Figures 4A,B). ThT fluorescence comparing reactions seeded with unenriched feces, enriched by large scale PAD-Beads, and NaPTA is shown in Figure 4. Based on these results, PAD-Bead enrichment was not further pursued for fecal samples, and NaPTA enrichment was used for all further experiments.

### Real-Time Quaking-Induced Conversion Detection of CWD Prions in Fecal Samples Collected From White-Tailed Deer in the Preclinical Stage of Prion Disease

**Figure 5:**
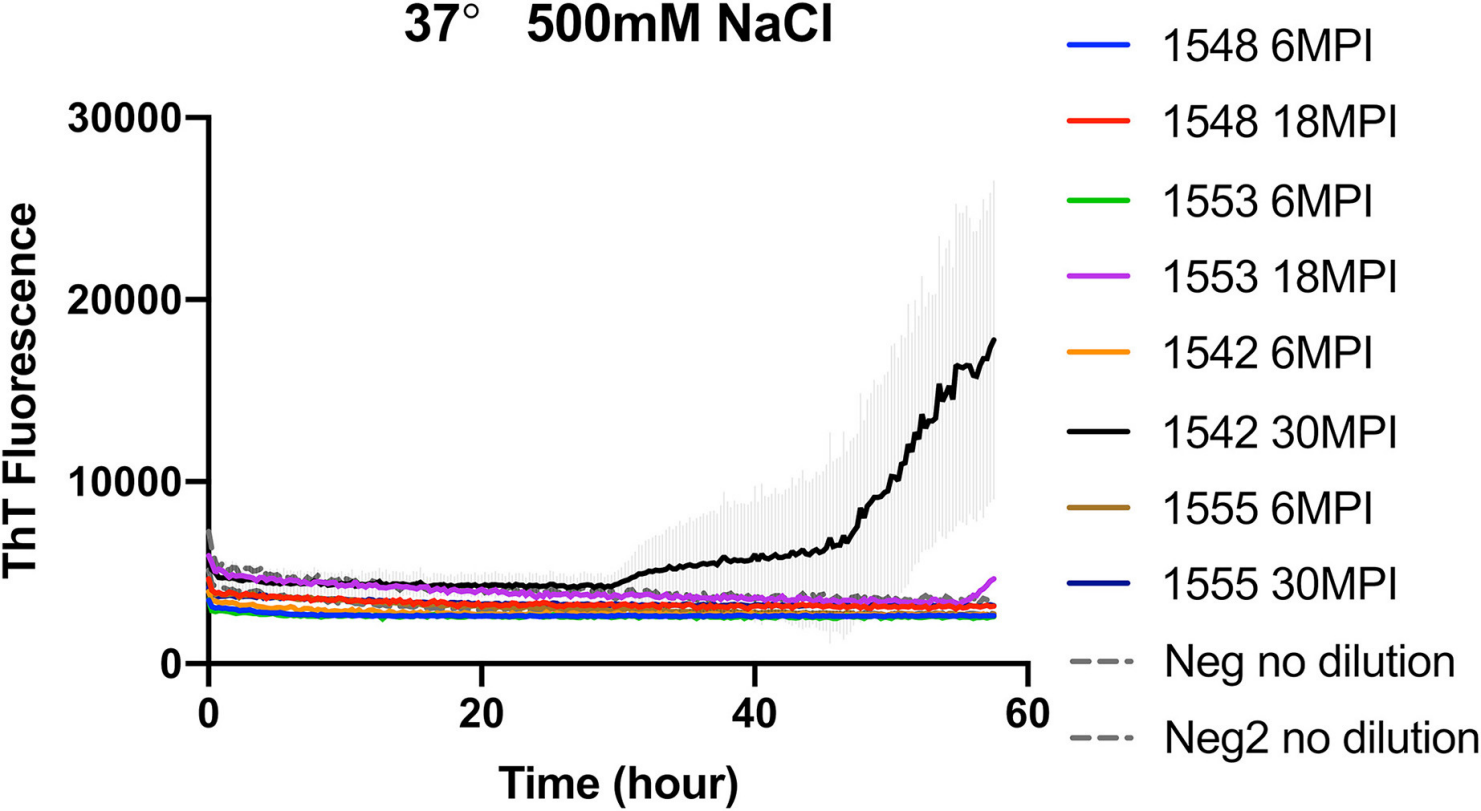
RT-QuIC reactions seeded with biopsy fecal homogenates from preclinical white- tailed deer. RT-QuIC reactions were seeded with 10^−1^ dilution of 10% fecal homogenate. All reaction mixtures contain 500 mM NaCl and measured at 37°C. Shown are the average ThT fluorescence readings determined from all replicates (four replicate reactions per each dilution).

**Figure 6:**
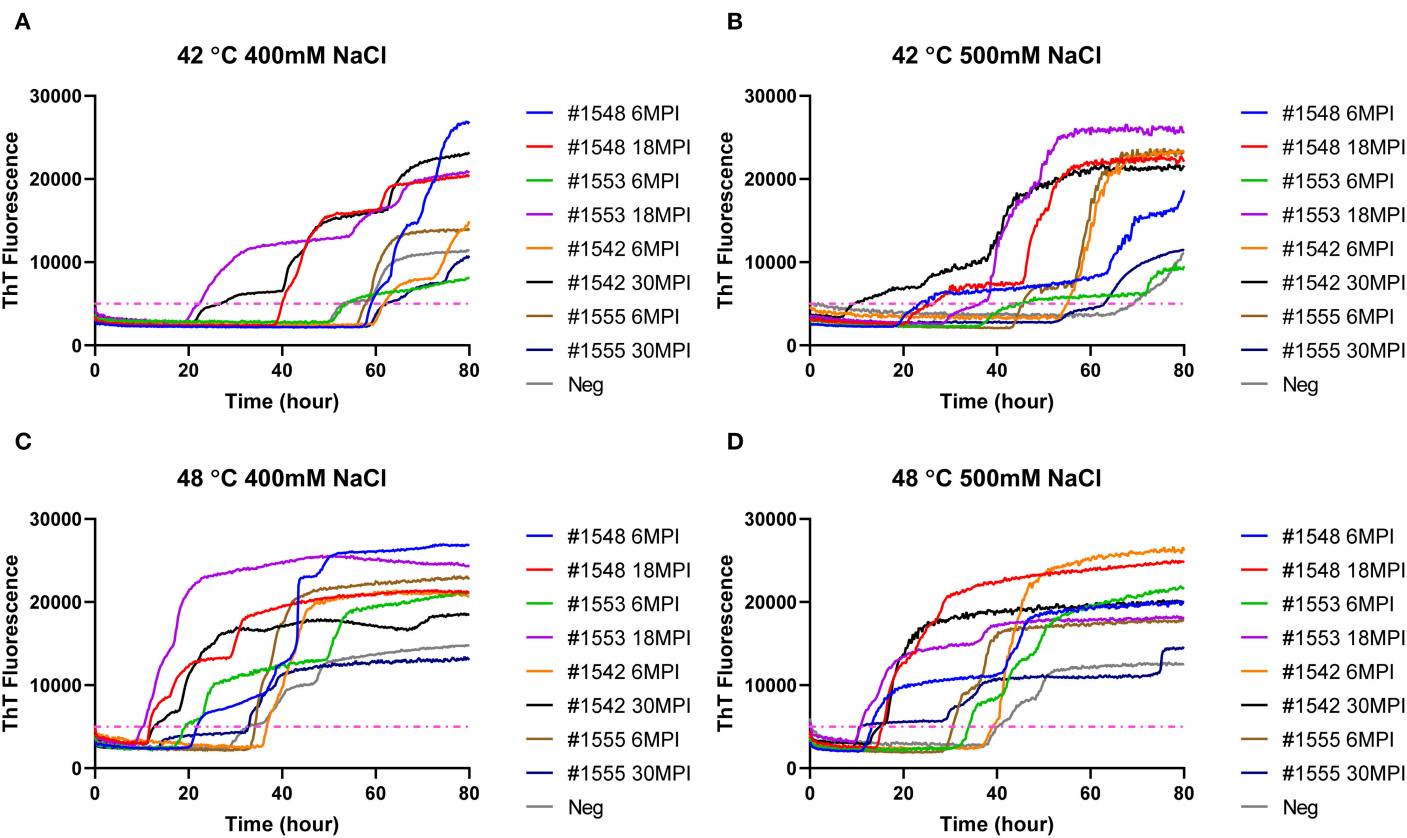
RT-QuIC reactions seeded with biopsy fecal homogenates from preclinical white- tailed deer. Reactions conditions for each sample are followed as **(A)** 400 mM NaCl at 42°C, **(B)** 500 mM at 42°C, **(C)** 400 mM at 48°C, and **(D)** 500 mM at 48°C. Shown are the average ThT fluorescence readings determined from all replicates (four replicate reactions per each dilution).

**Figure 7:**
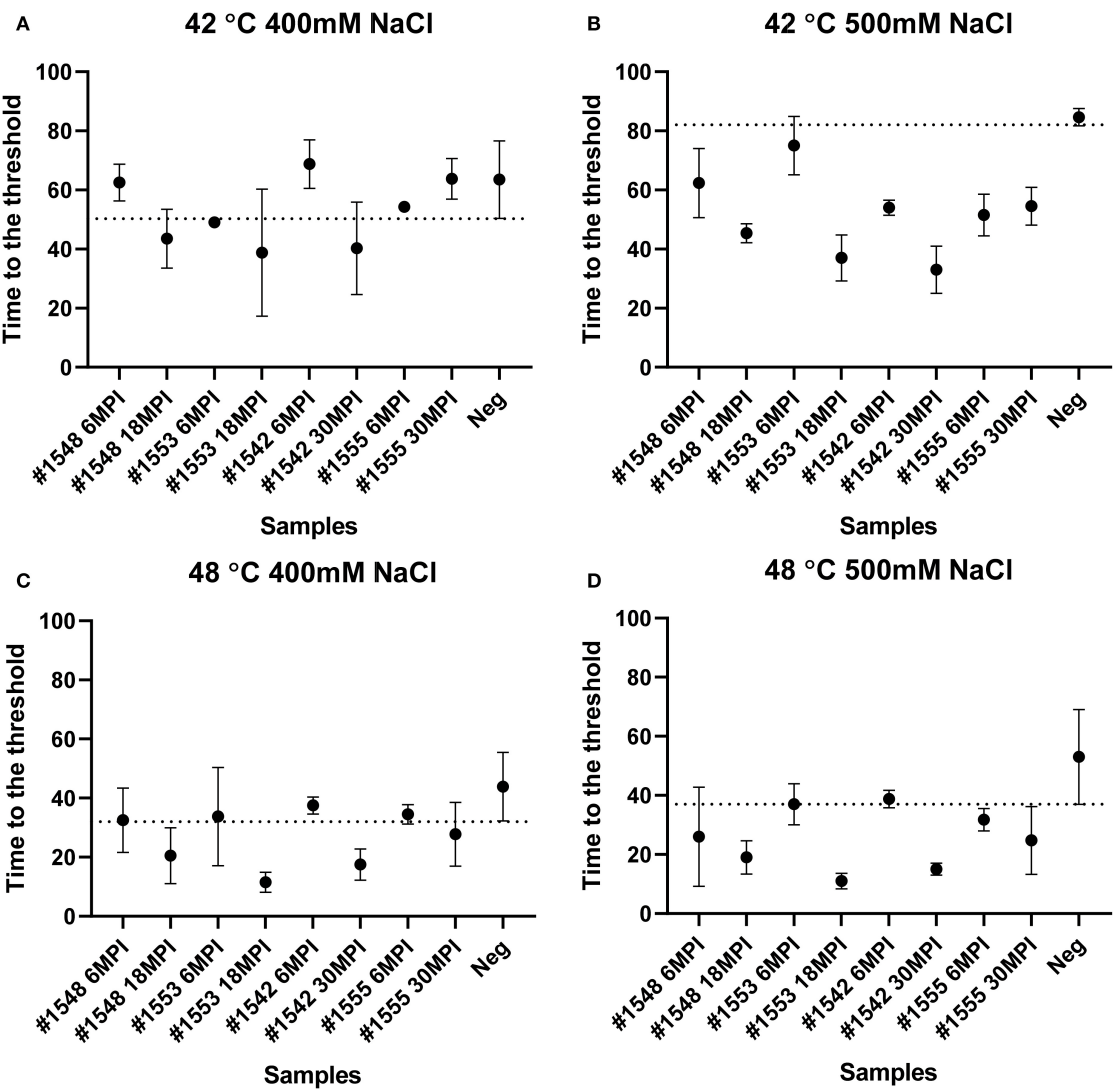
Lag time analysis of RT-QuIC reactions seeded with biopsy fecal homogenates from preclinical white-tailed deer. Reaction conditions for each sample are followed as **(A)** 400 mM NaCl at 42°C, **(B)** 500 mM NaCl at 42°C, **(C)** 400 mM NaCl at 48°C, and **(D)** 500 mM NaCl at 48°C. Circles represent the mean and bars represent SD of four replicate reactions. The horizontal line is the cutoff.

To evaluate if RT-QuIC assays can detect prions from fecal samples of preclinical white-tailed deer, fecal samples that were collected at times of routine animal handling were also seeded in RT-QuIC reactions. In total, eight fecal samples from four white tailed deer were collected. Four samples are from animal #1553 and #1548 after 6 and 18 months of inoculation, and another set of four samples received from #1542 and #1555 after 6 and 30 months of inoculation were used to run the assay. All fecal samples were NaPTA enriched and tested for prion detection in RT-QuIC, and assays were measured at 37°C in the presence of 500 mM NaCl (Figure 5). However, most assays did not show any increase of ThT fluorescence except the one seeded with #1542 (30 months post inoculation). With low detection under these conditions, assays were repeated at higher temperatures (42 or 48°C) using both 400 mM and 500 mM NaCl (Figure 6). This increased overall conversion as evidenced by increased ThT fluorescence (Figure 6). Assays run at 42°C in the presence of 500 mM NaCl showed the increase of ThT fluorescence within 60 h of lag time for most fecal sample collected from biopsy, and the reactions were well-separated from the assay seeded with feces of negative control animal (Figure 6B). Assays running at 48°C not only shorten the lag time by ~20 h for most fecal samples but it also shortens the lag time for negative control samples preventing the discrimination of positive samples from negative sample. Figure 7 shows the lag time for all seeding activity with a cutoff line for time to positive threshold such that positive samples are below the line. In terms of specificity, reactions under 42°C with 500 mM NaCl allowed the best results, and increasing temperature to 48°C improved the conversion efficiency but due to conversion in the negative control samples, the ability to discriminate positive from negative samples was not sufficient for use. It is worth noting that ThT fluorescence intensity from assays seeded with negative samples (Figure 6) is lower relative to the assays seeded with positive samples. For example, assays run at 48°C with 500 mM NaCl show higher ThT fluorescence intensity for positive samples compared to the intensity for negative samples.

### Sodium Iodide Reduces Lag Time in the Detection of CWD Prions in Fecal Samples Collected From White-Tailed Deer in the Preclinical Stage of Prion Disease

**Figure 8:**
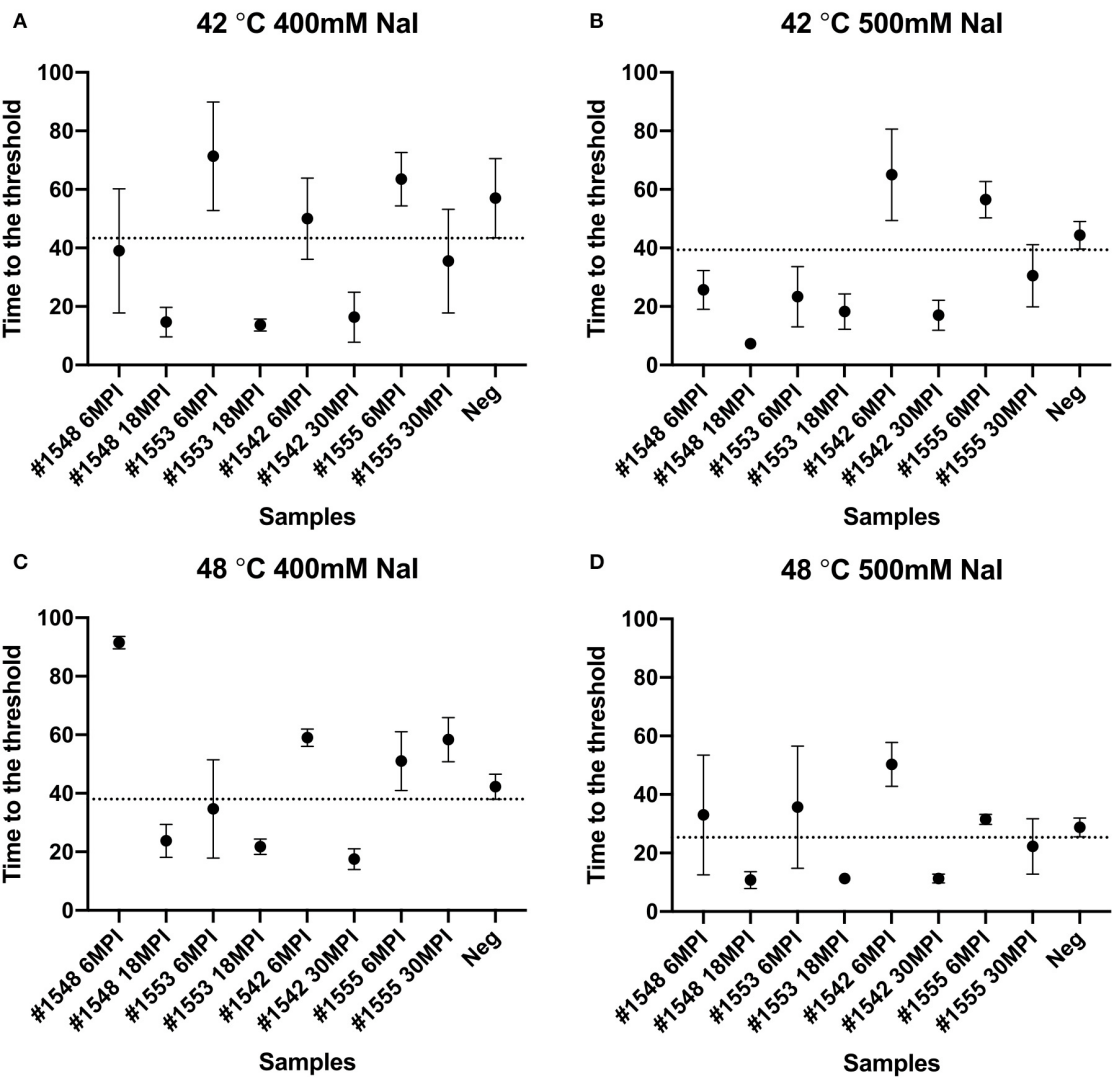
Lag time analysis of RT-QuIC reactions seeded with biopsy fecal homogenates from preclinical white-tailed deer in the presence of NaI. Reactions conditions for each sample are followed as **(A)** 400 mM NaI at 42°C, **(B)** 500 mM NaI at 42°C, **(C)** 400 mM NaI at 48°C, and **(D)** 500 mM NaI at 48 °C. Circles represent the mean and bars represent SD of four replicate reactions. The horizontal line is the cutoff.

**Figure 9:**
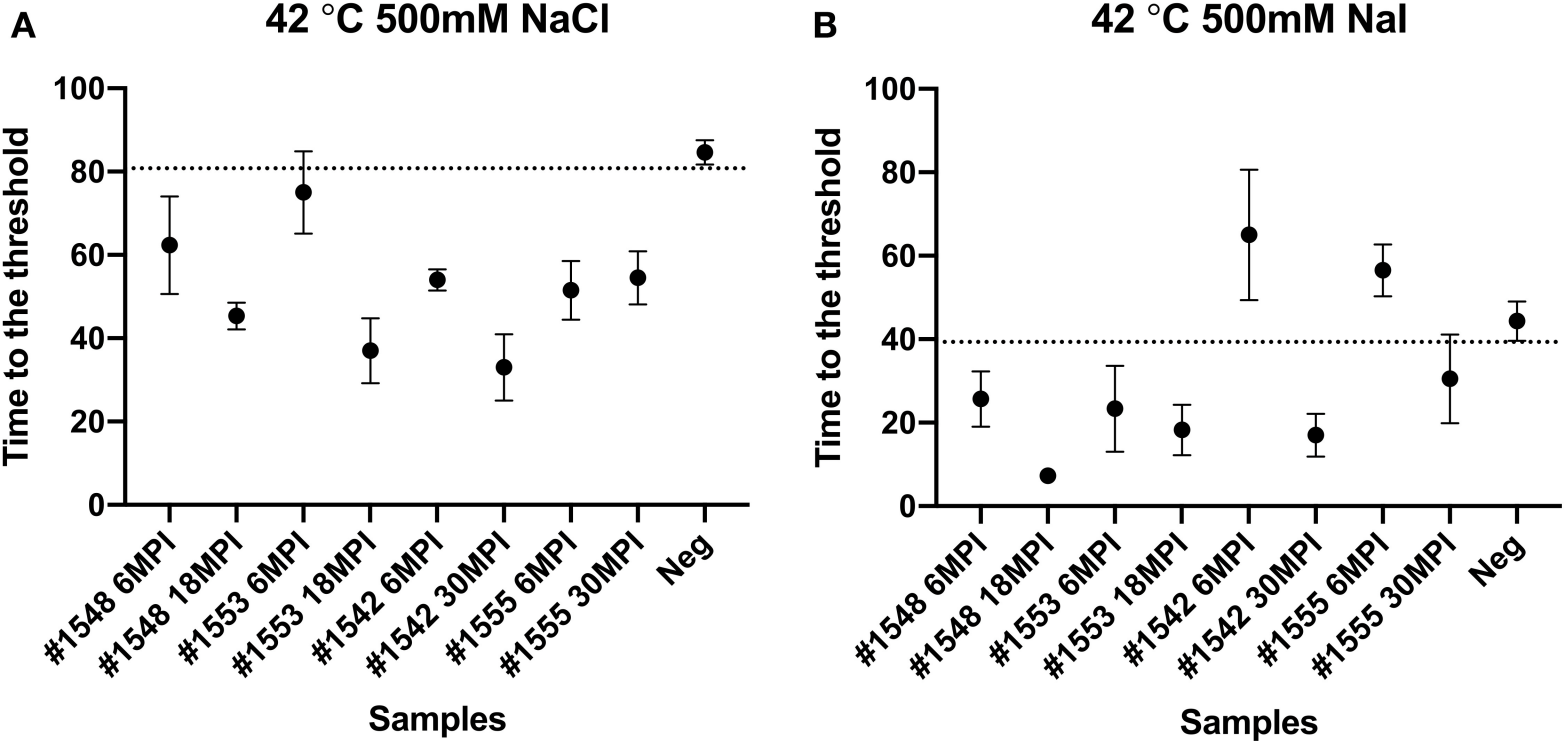
Comparison of lag time analysis of RT-QuIC reactions seeded with biopsy fecal homogenates from preclinical white-tailed deer in the presence of 500 mM NaCl **(A)** or NaI **(B)**. Circles represent the mean and the bars represent SD of four replicate reactions. The horizontal line is the cutoff.

We also tested another salt, NaI, in the reaction mixtures for detecting prions from fecal samples that were collected at times of routine animal handling. Instead of using NaCl, 400 or 500 mM of NaI was added in the RT-QuIC reaction mixtures and they were measured either at 42°C or 48°C. Overall, most reaction assays containing NaI showed a shorter lag time compared to the assays containing NaCl (Figure 8). Again, reactions performed at 48°C shorten lag time with NaI but the high temperature stimulated spontaneous reactions for negative control in early lag time. Among the reaction conditions, assays containing 500 mM NaCl measured at 42°C were chosen for optimal conditions to detect prions from biopsy fecal samples when considering both conversion efficiency and specificity. Figure 9 shows comparison between assays run with 500 mM NaCl or 500 mM NaI. In addition, Table 2 shows the lag time analysis for the comparison of all the assays containing NaCl and NaI in different temperatures and concentrations. Overall, the replacement of NaCl with NaI allowed us to detect PrP^Sc^ from fecal samples in short lag time but with a reduced separation from the negative control. This is most apparent in Figure 9 where two inoculated animals at six MPI were no longer differentiated from negative. This experiment indicates that NaCl is still the better salt choice for fecal samples using bank vole substrate since all fecal samples from inoculated animals exhibited ThT fluorescence indicative of fibril formation in a shorter time relative to the negative control samples.

**Table 2:** Lag time analysis from real-time quacking-induced conversion reactions seeded with preclinical fecal samples in different temperatures, different salt types, and concentrations.

	**#1548 6 MPI**	**#1548 18 MPI**	**#1553 6 MPI**	**#1553 18 MPI**	**#1542 6 MPI**	**#1542 30 MPI**	**#1555 6 MPI**	**#1555 30 MPI**	**Neg**
42°C 400 mM NaI	39.0 ± 21.2	14.7 ± 5.0	71.3 ± 18.5	13.7 ± 2.0	50.0 ± 13.8	16.3 ± 8.5	63.5 ± 9.1	35.5 ± 17.6	57.0 ± 13.6
42°C 500 mM NaI	25.7 ± 6.7	7.25 ± 1.9	23.3 ± 10.3	18.3 ± 6.0	65.0 ± 15.6	17.0 ± 5.1	56.5 ± 6.2	30.5 ± 10.6	44.3 ± 4.7
48°C 400 mM NaI	91.5 ± 2.1	23.8 ± 5.6	34.7 ± 16.8	21.8 ± 2.6	59.0 ± 2.9	17.5 ± 3.5	51.0 ± 10.0	58.3 ± 7.6	42.3 ± 4.3
48°C 500 mM NaI	33.0 ± 20.0	10.8 ± 2.9	35.7 ± 20.8	11.3 ± 1.0	50.3 ± 7.5	11.3 ± 1.5	31.5 ± 1.7	22.3 ± 9.5	28.8 ± 3.2
42°C 400 mM NaCl	62.5 ± 6.2	43.5 ± 9.9	49 ± 1.6	38.7 ± 21.5	68.7 ± 8.2	40.3 ± 15.6	54.3 ± 1.5	63.7 ± 6.9	63.5 ± 13.1
42°C 500 mM NaCl	62.3 ± 11.7	45.3 ± 3.2	75.0 ± 9.9	37.0 ± 7.8	54.0 ± 2.6	33.0 ± 7.9	51.5 ± 7.0	54.5 ± 6.4	84.6 ± 2.9
48°C 400 mM NaCl	32.5 ± 10.9	20.5 ± 9.5	33.8 ± 16.6	11.5 ± 3.4	37.5 ± 2.9	17.5 ± 5.3	34.5 ± 3.3	27.8 ± 10.8	43.9 ± 11.6
48°C 500 mM NaCl	26.0 ± 16.7	19.0 ± 5.6	37.0 ± 6.9	11.0 ± 2.6	38.7 ± 2.9	15.0 ± 2.0	31.7 ± 3.7	24.7 ± 11.4	53.0 ± 16.1

*All times were recorded in hours*.

## Discussion

In this study, we have optimized RT-QuIC reaction conditions to enhance the detection of PrP^Sc^ from fecal homogenates from clinical and preclinical white-tailed deer. Different factors including salt concentrations and temperature were tested with regard to sensitivity and specificity of detection of infectious prions from fecal samples using bank vole recombinant prion protein as the amplification substrate. For fecal samples collected from clinically affected deer, a reaction temperature of 37°C showed prion detection, as previously reported by Henderson and colleagues where they observed less spontaneous reactions by reducing reaction temperature from 42 to 37°C for fecal prion detection in RT-QuIC (8). In the data presented in this study, the assays seeded with fecal samples from clinically affected animals showed prion seeding activity at 37°C even without any enrichment. However, for fecal samples collected from preclinical deer inoculated with CWD, RT-QuIC conditions had to be further optimized to enhance the prion detection. Using high salt concentration like 500 mM NaCl and increasing temperature from 37 to 42°C enhanced the seeding activity of fecal samples and allowed for discrimination between assays seeded with positive fecal samples and assays seeded with negative controls. We also tested higher temperatures like 48°C for better sensitivity of fecal prions in RT-QuIC. Higher temperatures did shorten the lag time of seeding activity overall, however, assays performed at 48°C also shorten the lag time of assays seeded with negative control, which led to an inability to discriminate positive samples from negative samples. Modified conditions were identified that enhance detection of prions in fecal samples from preclinical animals. Further, it was ultimately determined that for RT-QuIC reactions seeded with fecal samples from preclinical animals, it is desirable to incorporate NaPTA enrichment prior to RT-QuIC for improved detection of seeding activity.

A previously published report that had applied different ions of the Hofmeister series of ions to RT-QuIC reactions showed enhanced sensitivity of seeding activity for assays seeded with ear homogenate from CWD infected deer in the presence of NaI instead of NaCl (28). Here, we compare the seeding activity of fecal samples in the presence of NaI to that of NaCl. As can be seen in Figure 8, similar result was observed for fecal samples as was previously reported for ear homogenate such that assays containing NaI had shorter lag times than assays containing NaCl. Assays seeded with negative sample also showed a shortened lag time for unseeded fibril formation under all reaction temperatures complicating discrimination of positive and negative samples. When we compare the results of RT-QuIC with NaI and NaCl, it is clear that NaI could be useful for quick detection with samples that have relatively high amount of prions. However, NaCl, despite the longer lag time, provided 100% sensitivity with better separation of positive assays from negative assays. This is in contrast to the previous report utilizing NaI where assays seeded with ear homogenate from CWD infected deer in the presence of NaI showed higher sensitivity and better specificity for prion detection than NaCl when used in RT-QuIC assays (28).

It is well-documented that fecal samples from cervids infected with CWD contain detectable prions (8–11). RT-QuIC seems practical to detect infectious prions from fecal samples of CWD infected cervids based on our study and previous reports. John and colleagues published a report that had shown RT-QuIC detection of fecal samples obtained from preclinical white-tailed deer. They tested fecal samples collected 20 and 30 months post inoculation, and they were only able to detect PrP^Sc^ from fecal sample collected at 30 months post inoculation possibly due to non-enrichment of the samples (11). Later, Cheng and colleagues showed that fecal prions of CWD infected elk could be detected with a NaPTA enrichment and detection by RT-QuIC using recombinant mouse prion protein (10). Given their choice of substrate and reaction condition, they found it necessary to replace the substrate after 24 h of RT-QuIC reaction in order to see better detection of fecal prions. Henderson and colleagues also published a report indicating that fecal prions could be detected by RT-QuIC using Syrian hamster recombinant prion protein substrate and iron-oxide bead extraction as an enrichment rather than NaPTA precipitation (8). They also evaluated the reaction temperature, 37, 40, or 42°C to reduce the lag time for detection of positive samples and decrease spontaneous fibril formation in negative controls finding 37°C to be optimal for preventing spontaneous reactions while still allowing good sensitivity. In this work, we used recombinant BV substrate which is generally reported as a universal substrate for detecting various prion diseases from both animals and humans by RT-QuIC (19) to detect CWD prions in fecal samples from white-tailed deer intranasal inoculated with CWD sourced from experimentally passaged through either white-tailed deer or racoon. Like Cheng and colleagues, we applied NaPTA precipitation for enrichment of prions in all fecal samples we collected. We also applied another enrichment methodology PAD-Beads, and has been previously reported for successfully enhanced RT-QuIC assays with brain homogenate of TSE inoculated animals (16). However, when PAD-Beads enrichment was applied for fecal samples, reactions with negative fecal samples were also seeded, which suggested that PAD-Beads may not be suitable for fecal sample enrichment coupled with RT-QuIC. Most notable in this work is that by 6 months post-inoculation, prion seeding is identified in both TSE isolates. For the samples included in this study, white-tailed deer CWD showed an incubation time in the 21–24 months range, while CWD passaged through raccoons prior to inoculation in white-tailed deer exhibited an onset of clinical signs in excess of 34 months with one of our samples not showing clinical signs at 56 months post inoculation. This highlights the potential for early clinical detection of TSEs using NaPTA enrichment coupled with RT-QuIC for the detection of CWD. Overall, combination of BV substrate, different salt concentration, and temperature allowed us to detect infectious prions within short lag time with good specificity from clinical or preclinical white-tailed deer.

Altogether, we confirm again that RT-QuIC is a powerful tool to detect infectious fecal prions from CWD infected white-tailed deer. Use of feces is a non-invasive and non-stressing approach to sampling of animals, of particular importance for non-domesticated animals that may be less tolerant to the handling required for sampling by other means. This is of importance to the management of both wild and farmed cervids and is also of use in experimental settings where repeated sampling of an individual animal would be otherwise difficult. Ultimately, fecal sampling may prove useful in the determination of disease prevalence in a geographic region or within a herd.

## Data Availability Statement

The original contributions presented in the study are included in the article/supplementary material, further inquiries can be directed to the corresponding author.

## Ethics Statement

The animal study was reviewed and approved by National Animal Disease Center Institutional Animal Care and Use Committee.

## Author Contributions

SH and EN: conceptualization, methodology, and writing original draft. SH: formal analysis and investigation. EN and JG: resources. EN: supervision. SH, EN, and JG: writing review and editing. All authors contributed to the article and approved the submitted version.

## Conflict of Interest

The authors declare that the research was conducted in the absence of any commercial or financial relationships that could be construed as a potential conflict of interest.
